# Mapping Human Health Outcomes in Relation to Harmful Algal Blooms Using a Translational Research Framework

**DOI:** 10.1093/geroni/igaf122.4159

**Published:** 2025-12-31

**Authors:** Rebecca Koszalinski, Malcolm McFarland, John Reif, Adam Schaefer, Michael Parsons, Ann Cary, Alex Rockenstyre, Rachael Shinbeckler

**Affiliations:** University of Central Florida, Orlando, Florida, United States; Florida Atlantic University, Fort Pierce, Florida, United States; Colorado State University, Fort Collins, Colorado, United States; Self, Richmond Hill, Georgia, United States; Florida Gulf Coast University, Ft Myers, Florida, United States; Florida Gulf Coast University, Ft. Myers, Florida, United States; University of Central Florida, Orlando, Florida, United States; Florida Gulf Coast University, Ft. Myers, Florida, United States

## Abstract

Aim: To evaluate Human Health Outcomes (HHO) after exposure to Harmful Algal Blooms (HABs) in aging adults through the National Institute of Environmental Science, Research Translational Framework (NIEHS-TRF). Our program of research required a cohesive framework for continued multidisciplinary collaboration (environmental sciences, epidemiology, & nursing) and research dissemination in healthcare literature. The initial research (2018) during a bloom detected cyanobacterial microcystins in the nares of 95% of aging adults (n = 125) while a different analysis (2023) indicated that residential and recreational exposures were significantly associated with increased risks of respiratory (74%), gastrointestinal (35%) and ocular symptoms (62%). A recent study (2025) affirmed that frequencies of reporting for these symptom groups were significantly higher during bloom periods than non-bloom periods. It became important to diagram our research trajectory. The process of cartography consisted of three steps on the NIEHS-TRF: 1) identification of research categories and activities (depicted visually by rings/nodes) that linked to research program outcomes, 2) within each category (visual ring), linked specific works and program outcomes to activities (visual nodes), and 3) coherently depicted visually as an overall map. The outcomes of the cartography process yielded a cohesive research trajectory, informed grant proposals, guided manuscripts and abstracts all focused-on understanding and addressing potential HHO of exposure to HABs in the aging adult population. Cartography using the NIEHS-TRF framework is recommended for nurse scientists and clinical researchers to support purposeful multidisciplinary collaboration and to strengthen planning and evaluation with clear visualization(s).

